# 

**Published:** 1910-12-17

**Authors:** 


					Publications.
By A. H. TUBBY, 1MT.S.
DEFORMITIES.
Six hundred pages, 300 Illustrations. Price 17s.
" Standard work on the subject in the English language."
British Medical Journal.
SURGERY OF PARALYSES.
By A. H. TUBBY, M.S.Lond., F.R.C.S.Eng., and
ROBERT JONES, F.R.C.S.E.
Illustrated by 93 figures and 68 cases. Pp. 301. 10s. net.
" Oan confidently recommend it as a practical summary of the most
modern views."?Edinburgh Medical Journal.
" Indicates original and successful methods of treatment."
American Journal of Orthopcedic Surgery.
London : Macmillan & Oo., Ltd. New York : The Macmillan Co.
Just Issued. Ninth Edition, Rewritten and Enlarged, 9s. net.
PHARMACY, MATERIA MEDIGA & THERAPEUTICS.
Being a Commentary on the B.P. and its Drugs, with their Preparations,
and on all the new Non-official Remedies in use, with Chapters on the
different processes used in Dispensing, Prescription-writing, Latin
Glossary, &c. By Sir W. WHITLA, M.D., LL.D., Professor, Materia
Medica, Queen's University, Belfast, and Senior Physician, Royal Victoria
Hospital.
By the same Author. New Work. 1,900 pages. Crown 8vo. 25s. net.
PRACTICE OF MEDICINE.
Containing in two Handy Volumes a complete and independent Article on
the Etiology, Symptoms, and Diagnosis of every Medical Disease; arranged
in Dictionary Form for Practitioners and Students.
London: BAiLLifcRE, Tindall & Cox, 8 Henrietta Street, W.C.
A NEW BOOK. Crown8vo.5s.net.
THE NEWER REMEDIES (a Practical Guide To).
By J. M. Fortescue-Brick dale, M.A., M.D. Oxon., Phys., Clifton
Coll.; Asst. Phys., B'tol Roy. Infy.; Lect. Pharm., Univ. of Oxford ; Clin.
Lect., Univ. of Bristol. An account of the Principal New Drugs, with
indications as to their value and employment.
" Will help the harassed practitioner."?Hospital.
" We ven cordially recommend it."?South African Medical Record.
Bristol: J. Wright & Sons, Ltd. London : Simpkin & Co., Ltd.
By Sir WILLIAM BENNETT, K.C.V.O., F.R.C.S.
RECENTLY PUBLISHED. Crown 8vo. 5s. net.
INJURIES AND DISEASES OF
THE KNEE JOINT.
With 34 Illustrations.
KISBET &; CO.
Also, 8vo. 6s. FOURTH EDITION.
MASSAGE AND MOVEMENTS IN
RECENT FRACTURES;
Sprains and their Consequences; Rigidity of
the Spine and the Management of Stiff Joints.
With 23 Illustrations.
Ii O IT (3- IMI .A. 1ST S , G-BEE1T &; CO.
THERAPEUTICS OF THE CIRCULATION
By Sir LAUDER BRUNTON, Bart., M.D., D Sc., F.R.C.P., <frc.,
Consulting Physician to St. Bartholomew's Hospital.
Published under the Auspices of the University of London.
With numerous Diagrams. Medium 8vo. 7s. 6d. net.
JOHN MURRAY, Albemarle Street, W.
FIFTH EDITION. With 50 Illustrations. 51- net.
THE SCHOTT METHODS OF THE TREATMENT
OF CHRONIC DISEASES OF THE HEART.
With an Account of the Nauheim Baths and of their Therapeutic Exercises.
By W. BEZLY THORNE, M.P., M.R.O.P.
London : J. & A. Churchill, 7 Great Marlborough Street.
Fractured Base.
Laceration* of the brain by contrecoup is more
common than direct laceration, and consequently
any decompression operation .should be conducted,
in case of doubt, on the side of the' head opposite to
that at which the injury was received.?Mr. L. B.
Rawling.
Infection of the Urinary Tract.
The commonest micro-organism is the colon
bacillus. In the absence of pus from the urine the
mere presence of a few B. coli does not necessarily
denote disease; this state of things is exceedingly
common both in adults and in children, and especially
in females. Both constipation and diarrhoea appear
to favour the appearance of the bacilli, and they
occur in the urine of a large proportion of all febrile
patients. The tubercle bacillus may occur alone, or
not infrequently as a mixed infection with staphylo-
coccus albus; indeed, the isolation of staphylococcus
albus from a purulent urine should always suggest
the possibility of a tuberculous infection. Tubercu-
losis of the urinary tract, or of the kidney substance,
is such a common condition that the urine should
be carefully searched for T.B. in any case of doubtful
nature in which symptoms are referable to these
structures.?Dr. T. J. Horder.
Appointments and Resignations.
The Committee of Management of the Infants Hospital,
Vincent Square, Westminster, has appointed W. E.
Robinson, M.D., to the poet of physicians' assistant.
Obituary.
The death is announced from Bradford of Mr. William
Henry Horrocks, F.R.C.S., senior surgeon to the Bradford
Royal Infirmary and a former president of the Bradford
Division of the British Medical Association. H? studied
at the University Colleges of London and Liverpool, and
at Owens College, Manchester, took his M.R.C.S. in 1882,
and obtained the Fellowship of the Royal College;' of
Surgeons by examination and the M.B. degree of the
University of London in 1886. He was awarded honours
in forensic medicine, physiology, and botany. His appoint-
ments included the post of senior demonstrator of anatomy
at University College, London, and of house surgeon and
ambulance surgeon at the Liverpool Northern Hospital.
He contributed to medical journals and was the author
of a Life of Sir Astley Cooper.
Professor Franz Konig, the former head of the surgical
Hospital of the Charite, Berlin, says the Times corre-
spondent, has died at the age of seventy-eight. He was born
at Rothenburg (Fulda), and his father was body physician
to the Landgraf of Hesse-Rothenburg. He studied surgery
under Professor Roser at Marburg, and then joined the
small hospital at Hanau. There he distinguished himself
by his surgical work so much that in 1869 he was appointed
Professor of Surgery at the University of Rostock. Later
he accepted a similar post at Gottingen, where he remained
for twenty years. In 1882 he was invited to succeed
Langenbeck at Berlin, but declined on grounds of ill-health.
Thirteen years later, on the death of Professor von Bard-
leben, the offer was again made to him?an unusual experi-
ence?and this time he accepted it. He continued to
hold the post until 1904. Konig's reputation was based
largely on his skill in the treatment of articular tuber-
culosis. His bust stands at the entrance to the new Klinik
in the Charite, in the building of which he took a great
interest.
Prince Alexander of Teck has received the following
additional contributions to the Prince Francis of Teck
Memorial Fund for the endowment of the Middlesex
Hospital: "A.," ?25; Messrs. Emile Erlanger and Co.,
?20; Lord Weardale, ?10 10s. ; Mrs. Halford, ?10; the
Hon. Sir W. A. C. Barrington, ?10; and Mr. and Mrs.
S. G. Asher, ?100.
3G6 THE HOSPITAL December 17, 1910.
APPOINTMENTS.
Dr. James Randal Hutchinson, M.B., Ch.B., D.P.H.,
senior assistant to the medical officer of health of
Manchester and medical officer of health for the
Withington district of Manchester, has been appointed
medical officer of Wigan at a salary of ?500 a year. Dr.
Hutchinson is lecturer in bacteriology and sanitary science
in the Municipal School of Technology, Manchester.
Previous appointments held by him were : Assistant to
the medical officer of health of Blackburn, house surgeon
to the Eye and Ear Infirmary, Liverpool, house surgeon at
the Manchester Royal Infirmary, senior resident medical
officer of the Chorlton Union Hospital, and resident
medical officer of the City Hospital, Clayton Yale, Man-
chester. He is thirty-one years of age.
Mr. Charles Dobell, J.P., has been re-elected Chair-
man, and Mr. John I. Barton, Deputy-Mayor of Ryde,
Vice-Chairman of the Isle of Wight Joint Hospital Board.
Mr. Herbert A. Johnson has been elected President of
the Derbyshire Hospital for Sick Children. He succeeds
three lady Presidents, the latest of whom was the Hon.
Blanche Curzon, now added to the list of Vice-Presidents
of the hospital. Mr. Reginald Boden has been elected to
the Board of Management, the following members being
again chosen : Mr. F. W. Greaves, Aid. Chambers, Mr.
H. Davis, Mr. H. M. Gray, Aid. W. Hart, Mr. H. M.
Hobson, Mr. N. J. Hughes-Hallett, Mr. T. H. Jones, Mr.
W. A. Wright, Mr. Gerald H. Smith, and Mrs. Hicks.
Mr. W. Campion has been re-elected Auditor. The follow-
ing were balloted for on the Elective Committee : Lord
Curzon, Mr. H. Gordon Ley, Mr. J. King, Mr. G. Argyle,
Aid. Woodiwiss, Mr. I. N. Woodiwiss, Mr. A. Swingler,
Mr. B. Stretton, Mr. H. A. Hubbersty, Mr. F. B. Wright,
Mr. R. Y. Dawbarn, and Mr. J. F. Thirlby.
Gifts and Bequests
An anonymous gift of ?100 has been received by the
Royal Hospital for Incurables, Putney Heath, in answer
to the appeal made bv Mr. Charles Dickens, which we
noticed at length on page 324 of The Hospital last week.
A Legacy of ?1,000 has been received by the Seamen's
Hospital Society from the late Mr. Wm. Bevan ; and a gift
of 200 garments for seamen has been received at the Dread-
nought Hospital, Greenwich, from the London Needlework
Guild.
Jottings.
Two Thousand Pounds are required before Decem-
ber 31 by the London Homoeopathic Hospital, Great
Ormond Street, W.C., to secure a promise of ?5,000.
Princess Marie Louise of Schleswig-Holstein pre-
sided last Saturday at the Royal Free Hospital, Gray's Inn
Road, at a meeting of the Princess Marie Louise Guild of
Work. She afterwards visited some of the wards and the
board-room, where an exhibition was held of the garments
provided by the Guild and by the members of the Ladies'
Association.
An entertainment consisting of Christmas carols as sung
and danced before the King in the presence at Whitehall
1640 will be held in the Town Hall, Spa Road, Ber-
mondsey, on Thursday and Friday, December 15 and 16,
at 6.30, in connection with the Guild of the Brave Poor
Things. Tickets, price 10s. 6d., 2s. 6d., and gallery Is.,
can be obtained at the door or by post from Mrs. C. W.
Kimmins, Dame Armstrong House, Harrow-on-the-Hill ;?
or Miss Alice Rennie, 36 Westbourne Terrace, Hyde Park,
W. The Guild gathers together all maimed people,
whether men, women, or children. Thus, when a society
provides a crutch, the Guild, with its socials and holidays,
sees that that crutch is put to the best use. In addition,
however, to the social work of the Guild Headquarters or
its various branches, education work is done at the Heritage
Craft Schools for Crippled Boys and Girls at Chailey,
Sussex.
We are informed that certain members of the honorary
medical staff of the Royal Infirmary, Newcastle, have re-
signed owing to a dispute as to the admission of patients.
The House Committee recently investigated certain points
in connection with the admission of patients into the
infirmary, and prepared a report upon the subject. The
members of the medical staff asked that this report should
be redrafted, but the Committee refused, and several mem-
bers of the staff sent in their resignations. A special'
meeting of the House Committee has been called to consider
the matter, with which we hope to deal in next week's
issue.
TO HOSPITAL SECRETARIES AND OTHERS.
We invite contributions from any of our readers, and
shall be glad to pay, according to length, for items of news
for the institutional section (including appointments, resig-
nations, gifts, etc.) which we may publish. All payments
for manuscripts used are made as early as possible after
the beginning of each quarter.
GENERAL PRACTITIONERS'
CONTRIBUTIONS.
The Hospital devotes a special page to General
Practitioners' contributions. We therefore invite
from practitioners contributions based upon their
experience in the management of cases, and in the
treatment and diagnosis of disease; especially shall
we be prepared to welcome articles dealing, practi-
cally, with treatment, and with the use and value
of new remedies and. methods.
No article should exceed 1,100 words, and, if
accepted, one guinea for a contribution of this
length will be paid to the writer after publication.
Each communication should be accompanied by a
stamped directed envelope for the return of the MS.
if found unsuitable.
Contributions should be written, or preferably
typed, on one side of the paper only, and all articles
sent in are accepted upon the distinct understanding
that they are forwarded to The Hospital only.
BUSINESS NOTICES.
Annual Subscriptions.
The rate of annual subscriptions to The
Hospital, either direct from the Office or through
agents, is: ?
For the United Kingdom  15s. a year.
For the Colonies and Abroad ... 17s. ,,
For the United Kingdom (with
Insurance Policy)  ?1 ,,
inclusive of postage in each case.
Letters relating to the Publishing, Sale and
Advertisement Departments must be addressed to
the Manager (not to the Editor): ?
The Manager,
The Hospital Building,
28 and 29 Southampton Street,
Strand, London, W.C.
THE SCIENTIFIC PRESS PUBLICATIONS.
A LEGAL HANDBOOK FOR THE USE
OF HOSPITAL AUTHORITIES.
By LEONARD TYER BRISTOWE, Barrister-at-Law.
2/6 post free.
MEDICAL HISTORY FROM THE EARLIEST TIMES.
By E. T. WITHINGTON, M.A.
Over 400 pp. Illustrated, 12/6 net, 12/10 post free.
HOSPITAL EXPENDITURE:
THE COMMISSARIAT.
For the Managers of Hospitals. 2/6 post free.
PHOTO-MICROGRAPHY.
By EDMUND J. SPITTA, M.R.O.S., F.R.A.S.
Demy 4to. with 41 Half-Tone Reproductions from original negatives and
63 Illustrations in the Text, 12/-.
FOR INSTITUTIONS.
CHARTS, CHART CASES, LEDGERS, ACCOUNT BOOKS, STOCK BOOKS, and all INSTITUTIONAL LITERATURE.
THE SCIENTIFIC PRESS, 28 & 29 Southampton Street, Strand, W.C.
THE HOSPITAL December 17, 1910.
THE IDEAL WORK OF REFERENCE.
SURDETT'S HOSPITALS & CHARITIES.
1910 EDITION.
The Year-Boole of" Philanthropy and Hospital Annual.
Edited by SIR HENRY BURDETT, K.C.B., K C.V.O.
Complete Guide and Directory of the Hospitals, Asylums, and Charitable Institutions throughout the English-speaking World-
Contains Special New Chapters on:?Voluntary Hospitals in 1909.?Soundness of the Voluntary System.?The need of
greater zeal on the part of those who accept office.?State-aided Hospitals in the U.S.A. and Canada.?The Appropriation of
Public Funds for the partial Support of Voluntary Hospitals in U.S.A. and Canada.?The Need of an Apostle and of greater
interest on the part of the Clergy, &c., &c.
TWENTY-FIRST YEAR OF PUBLICATION.
Over 1,000 pages. Price 10/6 net; Post Free, 10/11.
ORDER your copy of the Medical Whitaker NOW.
Publishers : THE SCIENTIFIC PRESS, Ltd, 28 & 29 Southampton Street, Strand, London, W.C.

				

